# A Novel Residual Dense Pyramid Network for Image Dehazing

**DOI:** 10.3390/e21111123

**Published:** 2019-11-15

**Authors:** Shibai Yin, Yibin Wang, Yee-Hong Yang

**Affiliations:** 1 Department of Economic Information Engineering, Southwestern University of Finance and Economics, Chengdu 611130, China; 2 Key Laboratory of Financial Intelligence and Financial Engineering of Sichuan Province, Southwestern University of Finance and Economics, Chengdu 611130, China; 3 Collaborative Innovation Center for the Innovation and Regulation of Internet-Based Finance, Southwestern University of Finance and Economics, Chengdu 611130, China; 4 Department of Engineering, Sichuan Normal University, Chengdu 610066, China; 5 Department of Computing Science, University of Alberta, Edmonton, AB T6G 2E8, Canada; herberty@ualberta.ca

**Keywords:** image dehazing, residual dense pyramid, encoder-decoder, convolutional neural network

## Abstract

Recently, convolutional neural network (CNN) based on the encoder-decoder structure have been successfully applied to image dehazing. However, these CNN based dehazing methods have two limitations: First, these dehazing models are large in size with enormous parameters, which not only consumes much GPU memory, but also is hard to train from scratch. Second, these models, which ignore the structural information at different resolutions of intermediate layers, cannot capture informative texture and edge information for dehazing by stacking more layers. In this paper, we propose a light-weight end-to-end network named the residual dense pyramid network (RDPN) to address the above problems. To exploit the structural information at different resolutions of intermediate layers fully, a new residual dense pyramid (RDP) is proposed as a building block. By introducing a dense information fusion layer and the residual learning module, the RDP can maximize the information flow and extract local features. Furthermore, the RDP further learns the structural information from intermediate layers via a multiscale pyramid fusion mechanism. To reduce the number of network parameters and to ease the training process, we use one RDP in the encoder and two RDPs in the decoder, following a multilevel pyramid pooling layer for incorporating global context features before estimating the final result. The extensive experimental results on a synthetic dataset and real-world images demonstrate that the new RDPN achieves favourable performance compared with some state-of-the-art methods, e.g., the recent densely connected pyramid dehazing network, the all-in-one dehazing network, the enhanced pix2pix dehazing network, pixel-based alpha blending, artificial multi-exposure image fusions and the genetic programming estimator, in terms of accuracy, run time and number of parameters. To be specific, RDPN outperforms all of the above methods in terms of PSNR by at least 4.25 dB. The run time of the proposed method is 0.021 s, and the number of parameters is 1,534,799, only 6% of that used by the densely connected pyramid dehazing network.

## 1. Introduction

The images taken on hazy days inevitably lose colour fidelity and intensity contrast, since floating particles in the atmosphere such as water droplets and dust particles absorb or scatter the light reflected from the scene object before it reaches the camera sensor. The degradation of sensor image quality introduces challenges to computer vision tasks such as video surveillance, aerial photography systems, image segmentation, and so on, all of which strongly rely on the quality of sensor images to realize subsequent visual tasks [1,2,3,4,5]. Hence, image dehazing is a very important problem and has created a wide range of interest in the research community.

In the past, many methods have shown outstanding performance in image dehazing [6,7,8]. Some of them are based on the following physical model:

(1) I(x)=J(x)t(x)+A(1−t(x)),

where *x* is the pixel location in the image. *I* represents the observed hazy image. *J* denotes the unknown clear image. *A* corresponds to the atmospheric light, and *t* denotes the scene transmission map indicating the portion of light that reaches the camera sensor. Assuming that the haze is homogeneous, we can further denote the transmission map t(x) as $e^{-\beta d(x)}$, where *d* represents the scene depth and β is the scattered coefficient of the atmospheric light. Since only the observed image *I* is known, estimating *J* from Equation (1) is a challenging ill-posed problem.

Conventional methods use hand-crafted features to capture the statistical properties of hazy images for estimating the transmission maps and atmospheric lights. For example, Tan et al. obtained a clear image by maximizing the per-patch contrast based on the prior that hazy images usually have lower contrast than that of clear images [9]. However, the halo artefacts and colour distortion usually appear in the dehazing results. He et al. estimated the transmission map via the dark channel prior (DCP), which states that each locally small patch of a haze-free image contains at least some pixels with very low intensities in one colour channel [6]. However, the method still has a challenge in recovering the real colour of white objects from the foreground. Tang et al. estimated the transmission map by searching the best combination of haze related features via random forest (RF) [10]. Due to the over-fitting problem of RF, this method cannot obtain favourable results. Using a colour attenuation prior (CAP), Zhu et al. modelled the depth of a hazy scene, but the parameters of the model were learned using supervised methods [11]. Fattal et al. discovered that the intensities of pixels in local image patches present a one-dimensional distribution in the RGB colour space [12]. Unfortunately, this strategy may lead to incorrect patch classification. Berman et al. further investigated the colour consistency theory of [12] and proposed a non-local prior (NLP), which asserts that a small number of distinct colours in a haze-free image can be well approximated by corresponding colour-lines in the RGB colour space [7]. However, this prior can be inaccurate in very hazy areas. To improve the robustness, an information fusion strategy and superior optimization methods are adopted in image dehazing methods. Yu et al. proposed a pixel-wise alpha blending method (PWAB) for predicting the transmission map, which blends the transmission maps estimated by the dark channel prior and the bright channel prior effectively [13]. Galdran et al. proposed artificial multi-exposure image fusion (AMEF) to estimate the clear image, which fuses multiply exposed images by a multi-scale Laplacian blending scheme [14]. Beltran et al. presented a novel genetic programming estimator (GPE) to predict the transmission map by finding optimal operators that can approximate the real results [15]. However, we found that the halo artefacts and colour distortions still occur when the fusion strategy and the optimization method fail to dehaze in a complex scene.

Recently, deep learning methods, especially those based on convolutional neural networks (CNNs), have been successfully applied to many computer vision and image processing tasks, e.g., semantic segmentation, image classification, object detection, and so on [16]. Due to their superior performance, CNNs are also used to solve the image dehazing problem. In general, image dehazing can be implemented by two kinds of CNN based methods, namely hybrid dehazing methods and end-to-end dehazing nets. Early approaches focussed on developing a hybrid strategy to remove haze using CNNs to estimate the transmission maps and using conventional methods to obtain atmospheric lights, e.g., the Dehazenet proposed by Cai et al. [17] maps a hazy image to a transmission map and uses empirical rules to acquire the atmospheric light. The cardinal colour fusion multi-scale CNN (C^2^MSNet) presented by Dudhane et al. predicts the transmission map by generating the multi-channel depth maps first [18]. One obvious problem of these methods is that they only focus on the estimation of transmission maps. If the transmission maps were not accurately predicted, they would impact negatively the final dehazed results. To avoid this problem, some works focus on developing end-to-end network architectures that can learn a direct mapping between a hazy image and its corresponding clear image. For example, Li et al. propose the all-in-one dehazing network (AODN) to predict the clear image of a scene from its hazy image without requiring estimating the transmission map [19]. Later, Zhang et al. proposed a densely connected pyramid dehazing network (DCPDN), which jointly learns the transmission map, atmospheric light and dehazing result by the pyramid densely connected network (PDCN), U network and generative adversarial network (GAN). Qu et al. proposed the enhanced pix2pix dehazing network (EPDN), motivated by the success of the generative adversarial network (GAN) [20]. Dudhane et al. further presented a generative adversarial networks with residual inception module (RIGAN) to remove haze [21]. Ren et al. proposed a gated fusion network (GFN) to estimate a clear image by fusing three derived images of the original hazy image effectively [22]. Based on GFN, Liu et al. further propose the GridDehazeNet (GridDN) for image dehazing, which adopts a pre-processing module to convert a hazy image to several derived images for information fusion and introduces a post-processing module for improving the quality of the clear image [23]. Although these methods integrate intermediate processing steps into one pipeline conveniently, the performance and architecture of these networks are still limited by the following factors: 1. Most end-to-end dehazing models are designed based on the deep encoder-decoder architecture with a large number of network parameters, e.g., DCPDN has 12,446,386 parameters, which not only consumes much GPU memory, but also is hard to train from scratch. 2. Existing methods neglect the structural and contextual information in all intermediate layers, leading to inaccurate dehazing results [24,25]. To alleviate the problem of parameters, the progressive image deraining network (PIDN) adopts a recursive residual structure to share the parameters in the network [26]. To extract more structural information in the intermediate layers, Liu et al. designed a Simple Pooling-based Network (SPN) for salient object detection by inserting the pyramid pooling module in each layer of the decoder [27]. The strategies borrowed from other vision models may give inspiration for image dehazing. However, improvements may not be achieved in dehazing due to the difficulty in applying the recursive module to the encoder-decoder network, and the encoder does not capture the required structural information.

**Figure 1:**
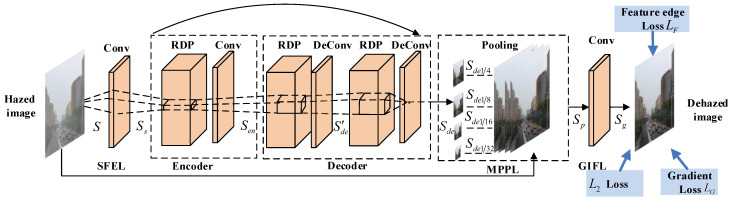
Overall architecture of the proposed residual dense pyramid network (RDPN) model.

**Figure 2:**
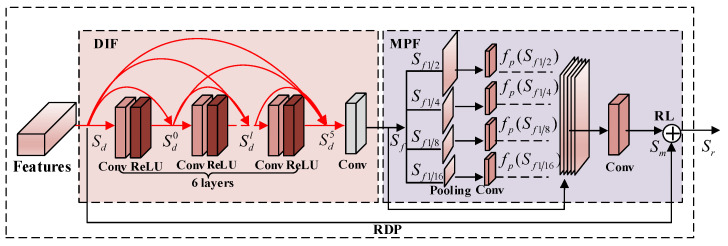
Residual dense pyramid (RDP) architecture.

In this work, we construct a light-weight network that captures more structural information from the intermediate layers. Therefore, the proposed end-to-end trainable residual dense pyramid network (RDPN) (Figure 1) can fully make use of structural information at different resolutions of all the layers with our new residual dense pyramid (RDP) (Figure 2). Generally speaking, our network is designed based on the encoder-decoder architecture, where the encoder contains one RDP and the decoder has two RDPs. For extracting features effectively from this light-weight network, the building module RDP is carefully designed with dense information fusion (DIF), multiscale pyramid fusion (MPF), and residual learning (RL), which not only combines the advantages offered by the popular dense connection and residual learning, but also leverages MPF to learn the structural information at different resolutions from each RDP.

The four main contributions of our work are summarized below:

- We propose a new end-to-end residual dense pyramid network (RDPN) based on the encoder-decoder architecture, which achieves high performance in image dehazing.
- We propose the residual dense pyramid (RDP) as the basic building module, which not only can effectively boost network performance by improving the information flow via dense connection and the residual learning mechanism, but also can learn structural features at different resolutions from all the layers of the encoder and decoder.
- By using one RDP in the encoder and two RDPs in the decoder, the light-weight RDPN contains much fewer network parameters (only 6% of that used by DCPDN [24]) and is much faster than existing CNN based methods (run time is reduced to 0.021 s).
- To enhance the generalization ability of the RDPN, both indoor and outdoor images are collected to generate a new synthetic dataset for training. The extensive experimental results demonstrate that our light-weight RDPN can achieve competitive results compared to other heavy-weight network models.

## 2. Residual Dense Pyramid Network for Image Dehazing

### 2.1. Network Structure

Inspired by the flexibility of the encoder-decoder network that can produce compelling results for image denoising, super-resolution and image harmonization, we explored the effective design of encoder-decoder in image dehazing. In most existing encoder-decoder modules, the dense block is employed as the basic building model and stacked layer-by-layer in a greedy fashion to construct the network architecture for feature transformation.

**Figure 3:**
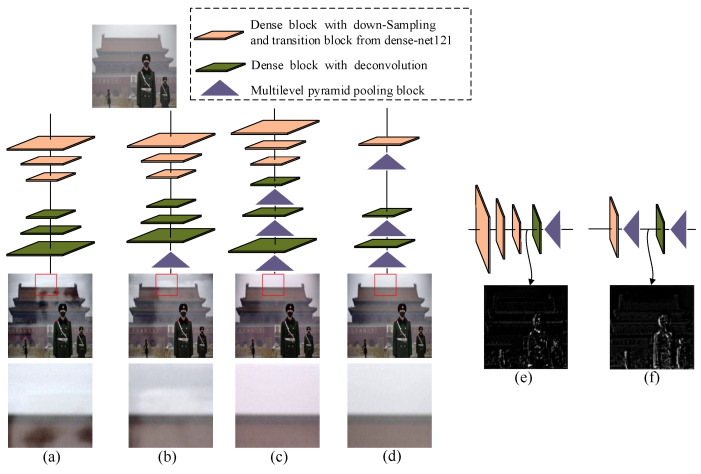
Comparison of different models. (**a**) Basic encoder-decoder network. (**b**) Encoder-decoder network with a multilevel pyramid pooling block in the end. (**c**) Encoder-decoder network with multilevel pyramid pooling blocks in the decoder. (**d**) Encoder-decoder network with multilevel pyramid pooling blocks in the encoder and decoder. (**e**) Visualizations of feature map at bottleneck of (**c**). (**f**) Visualizations of feature map at bottleneck of (**d**). All the models are trained on our synthetic 8000 hazy images. The real hazy image is shown at the top. Dehazing results with magnified details marked by red rectangles are displayed under each model in (**a**–**d**).

Consider Figure 3a as an example: three dense blocks with its down-sampling and transition blocks from dense-net121 [28] are used for building the encoder and the symmetrical dense blocks with corresponding deconvolutions as the decoder. Although this design utilizes the dense information flow to extract features with smaller sizes and transform them back to haze-free image, multi-scale structural information, which has been demonstrated to be effective in the traditional dehazing method, is totally neglected [24,25]. After inputting a real hazy image, the dehazing result has a halo effect (see the magnified detail in Figure 3a). Based on this model, Zhang et al. added a multilevel pyramid pooling block (MPPB) with pooling size $\frac{1}{32}$, $\frac{1}{16}$, $\frac{1}{8}$ and $\frac{1}{4}$ at the end of the decoder (see Figure 3b) and denoted this network as PDCN. Then, the global structural information with different scales was used to estimate the final result [24]. However, this scheme only takes the multi-scale information of the last layer into account, and high level global information from intermediate layers is not considered at all. The halo effect is not completely removed (see the white shadow in the magnified detail of Figure 3b). To further make full use of long range global information, some work, e.g., [27], adds MPPB into each layer of the decoder (see Figure 3c) to address the drawback of the model in Figure 3b. The improved model can remove the halo effect and enhance fine features, but the multi-scale information of the bottom-up pathway in the encoder is not explored. The colours in the dehazing result shift from the real colours. For example, the magnified detail in Figure 3c has turned to a reddish tint colour. In our proposed approach, we adopted the MPPB in the encoder. To verify the effectiveness of MPPB, only one dense block from dense-net121 [28] and one MPPB from PDCN [24] were used for building the encoder. Similarly, two MPPBs from PDCN [24] and two dense blocks with deconvolutions from dense-net121 [28] were adopted for constructing the decoder (see Figure 3d). In the corresponding output, we found not only that the halo effect was removed completely, but also the scene colours were much closer to the ones in the real world (see the output and magnified detail in Figure 3d). To understand this better, we further visualize the feature map at the bottleneck of the encoder-decoder framework. Figure 3e displays the intermediate feature map of Figure 3c. Meanwhile, Figure 3f shows the intermediate feature map of Figure 3d. By comparing these two results, we found that even one MPPB in the encoder allowed the network to obtain more global structural information than stacked dense blocks. For example, the edge and contour information is richer in Figure 3f than that in Figure 3e.

Based on the above discussion, we propose the residual dense pyramid (RDP) as the basic building module, which includes a dense block, residual learning and an MPPB. By building the encoder and decoder with RDP, the novel residual dense pyramid network (RDPN) can learn and fuse structural information from different resolutions at all layers. Generally speaking, RDPN takes a hazy image as input and predicts its corresponding dehazed result as output. As shown in Figure 1, the architecture of the RDPN mainly consists of five parts: a shallow feature extraction layer (SFEL), an encoder, a decoder, a multilevel pyramid pooling layer (MPPL) and a global information fusion layer (GIFL). Simultaneously, the skip connection with the same filter size is used to ease the training of the RDPN. The specific operations of these five parts are described in the next five subsections.

Shallow feature extraction layer (SFEL): As reported in many previous approaches [29], the low-level features such as contours and edges extracted in shallower stages usually have a smaller feature size and provide rich and detailed global information for deeper stages, which contain high-level features. Therefore, the SFEL was necessary for the encoder decoder network. Besides, in our work, SFEL also acts as a transition layer, which enabled the features with different spatial sizes to be captured gradually, avoiding shrinking the input with smaller spatial size sharply. In our design, we used a 1 × 1 convolution layer with a stride of two for extracting shallow features $S_{s}$. With half of the input image size, the shallow features not only can preserve the primary contours and edges for deeper stages, but also can suppress noise and unimportant details. The related operation can be expressed as:

(2) $$S_{s}=f_{SFEL}\left(S\right),$$

where *S* is the input image. $f_{SFEL}\left(\cdot \right)$ denotes a 1 × 1 convolution operation of SFEL. $S_{s}$ is the output and also serves as the input to the subsequent encoder.

Encoder: The encoder further extracts a set of feature maps from $S_{s}$. Inspired by dense connection [28] and residual learning [30], the proposed pyramid fusion can fully learn spatial information at different resolutions. In this paper, we propose the residual dense pyramid (RDP) as the basic building module and employ one RDP to construct the encoder. For each RDP, a 1 × 1 convolution layer is also used to capture all extracted features of the RDP and to enable down-sampling simultaneously. The output of the encoder can be formulated as:

(3) $$S_{en}=f_{{Conv}_{1}}\left(f_{RDP}\left(S_{s}\right)\right),$$

where $f_{RDP}$ denotes the composite function of our RDP, such as dense information fusion, multiscale pyramid fusion and residual learning. $f_{{Conv}_{1}}$ denotes the convolution operation following the RDP, and $S_{en}$ is the output. More details of the RDP are given in Section 2.2.

Decoder: After the features are extracted by the encoder, the decoder is utilized to restore the image content and reconstruct the dehazed image. In the proposed decoder, two RDPs are used. Similar to the encoder, two 1 × 1 deconvolution layers are used for each RDP to refine mapping features and to realize up-sampling. The decoder function can be described as follows:

(4) $$\begin{array}{c} S_{de}^{\prime }=f_{DeConv_{1}}\left(f_{RDP}\left(S_{en}\right)\right),S_{de}=f_{DeConv_{2}}\left(f_{RDP}\left(S_{de}^{\prime }\right)\right), \end{array}$$

where $f_{DeConv_{1}}$ and $f_{DeConv_{2}}$ denote two 3 × 3 deconvolution functions. $S_{de}$ is the final output of the decoder network.

Multilevel pyramid pooling layer (MPPL): Similar to [24], where features at different scales in an image are utilized for image dehazing, we also adopted a multilevel pyramid pooling layer to make sure that the features from a hierarchical global context prior were embedded in the resulting image, containing information from different scales. Here, four pooling operations with pyramid size 1/4, 1/8, 1/16 and 1/32 are employed to obtain multilevel features. Subsequently these pooled features are up-sampled to the size of the input image by nearest neighbour interpolation, followed by a concatenated operation with the original input to capture more global context information. The above operation can be expressed as:

(5) $$S_{p}=\left[f_{u}\left(S_{{de}_{1/4}}\right),f_{u}\left(S_{{de}_{1/8}}\right),f_{u}\left(S_{{de}_{1/16}}\right),f_{u}\left(S_{{de}_{1/32}}\right),S\right],$$

where $S_{{de}_{1/4}}$, $S_{{de}_{1/8}}$, $S_{{de}_{1/16}}$ and $S_{{de}_{1/32}}$ are the pooling results of $S_{de}$ with pyramid sizes 1/4, 1/8, 1/16 and 1/32, respectively. $f_{u}$ is the up-sampling operation and $S_{p}$ is the output of the MPPL.

Global information fusion layer (GIFL): The extracted global hierarchical features from the MPPL were further fused in the GIFL. In particular, we placed a 3 × 3 convolution layer in the last stage of the RDPN. The reconstructed result $S_{g}$ from the final convolution function $f_{GIFL}$ is given by:

(6) $$S_{g}=f_{GIFL}\left(S_{p}\right).$$

### 2.2. Residual Dense Pyramid

Motivated by the performance of the RDB, which combines the advantage offered by the dense block and the residual block, we propose a new compact block named RDP. Different from the existing RDB, we also added multiscale pyramid fusion in the RDP, which adopted the multiscale pyramid fusion to enable learning local context information and exploring the spatial relation in the RDP. The proposed RDP is shown in Figure 2, and all the components are discussed in subsequent subsections.

Dense information fusion design (DIF): Based on the observation that dense connections can maximize the information flow, we adopted dense connections as the basic structure in the RDP. As displayed in Figure 2, $S_{d}$ and $S_{o}$ denote the input and output features, respectively. Red coloured arrows indicate the dense connections between six convolution layers. Suppose the input feature number of $S_{d}$ is $G_{0}$ and the growth gate for dense connections is *G*, then $S_{0}$ has $G_{0}+5G$ feature maps. Although the higher growth rate *G* can introduce more local features, it also makes the network hard to train. Hence, it is necessary to reduce the number of features. Inspired by the RDB, to control and fuse the information flow, a 1 × 1 convolution layer indicated by grey colour shown in Figure 2 was added. The overall structure of our proposed DIF is specified as:

(7) $$\begin{array}{c} S_{f}=f_{DIF}(\left[S_{d},S_{d}^{0},\ldots ,S_{d}^{l},\ldots ,S_{d}^{5}\right],where,S_{d}^{l}=f_{D}(\left[S_{d},S_{d}^{0},\ldots ,S_{d}^{l-1}\right],0\leq l\leq 5. \end{array}$$

Here, $\left[S_{d},S_{d}^{0},\ldots ,S_{d}^{l-1}\right]$ defines the concatenation of the features produced using input $S_{d}$ and the preceding dense layers 0…l−1. $f_{D}$ denotes a 3 × 3 convolution function to produce *G* feature maps following the $l^{\mathrm{th}}$ layer and has $G_{0}+\left(l-1\right)\times G$ input feature maps. $f_{DIF}$ denotes a 1 × 1 convolution function to fuse the original input $S_{d}$ and outputs $S_{d}^{0},\ldots ,S_{d}^{5}$ from six dense connection layers into $S_{f}$ with *G* feature maps.

Multiscale pyramid fusion (MPF): Even though the DIF improved the information flow to a large extent, the features from DIF still lost the spatial relation. The local context information at different scales is helpful in this regard to explore spatial information at different resolutions. To address this problem efficiently, we used multiscale pyramid fusion (MPF). MPF was realized by four pooling operations and five convolution operations, as illustrated in Figure 2. In particular, we first pooled the feature maps from DIF into four different scales, such as 1/2, 1/4, 1/8 and 1/16. A single 1 × 1 convolution layer was introduced in the last stage for learning context information. The operation of MPF can be defined as:

(8) $$\begin{array}{c} S_{m}=f_{MPF}(\left[f_{p}\left(S_{f_{1/2}}\right),f_{p}\left(S_{f_{1/4}}\right),f_{p}\left(S_{f_{1/8}}\right),f_{p}\left(S_{f_{1/16}}\right),S_{f}\right], \end{array}$$

where $f_{p}\left(S_{f_{1/2}}\right),f_{p}\left(S_{f_{1/4}}\right),f_{p}\left(S_{f_{1/8}}\right),f_{p}\left(S_{f_{1/16}}\right)$ are the pooling results of $S_{f}$ with pooling sizes 1/2, 1/4, 1/8, 1/16, respectively. $f_{p}$ denotes a convolution operation for upsampling. $f_{MPF}$ refers to the final convolution function. $\left[f_{p}\left(S_{f_{1/2}}\right),f_{p}\left(S_{f_{1/4}}\right),f_{p}\left(S_{f_{1/8}}\right),f_{p}\left(S_{f_{1/16}}\right),S_{f}\right]$ represents the concatenation of pooling results and the original input. $S_{m}$ is the final output of the MPF.

Residual learning (RL): In order to enhance the RDP representation ability and to achieve better performance, we introduced the residual learning mechanism in the RDP before the final output. The final output $S_{r}$ of the RDP is defined as:

(9) $$S_{r}=S_{d}+S_{m}.$$

This motivation for this design was that the final RL can ensure that the RDP makes full use of the advantages offered by the DIF, MPF and RL and to enable high quality estimation of dehazed images.

### 2.3. Loss Function

Since earlier works demonstrated that the Euclidean loss $\left(L_{2}\right)$ easily leads to colour distortion or halo artefacts in the dehazed images [24], the works in [24,25] attempted to solve this problem by adding some edge preserving information in the loss, such as the combination of three losses, including the feature edge loss $L_{F}$, the gradient loss $L_{G}$ and standard $\left(L_{2}\right)$ loss function. For fair comparison, we adopted the same combined loss function for learning the parameters of the proposed RDPN. Considering that the combined loss function used in [24] was designed for the U net, PDCN and GAN jointly, we only adopted part of the loss function that is used in PDCN.

(10) $$L=L_{2}+\lambda L_{G}+\beta L_{F},$$

where λ and β are weighting coefficients for loss terms $L_{G}$ and $L_{F}$, respectively. Let $I_{i},i=1,2\ldots ,N$ and $J_{i},i=1,2,\ldots ,N$ represent the set of hazy images and the set of corresponding ground truths, respectively. Then, $L_{2}$ is defined as:

(11) $$L_{2}=\frac{1}{N}\sum_{i=0}^{N}{∥f\left(I_{i},\Theta \right)-J_{i}∥}_{2},$$

where *f* represents the proposed dehazing network and Θ denotes the parameters in *f*.

The gradient loss $L_{G}$ is defined using gradient operations of the horizontal and vertical directions:

(12) $$L_{G}=\frac{1}{N}\sum_{i=1}^{N}{∥(G_{v}(f\left(I_{i},\Theta \right)-G_{v}\left(J_{i}\right)∥}_{2}+\frac{1}{N}\sum_{i=1}^{N}{∥(G_{h}(f\left(I_{i},\Theta \right)-G_{h}\left(J_{i}\right)∥}_{2},$$

where $G_{v}$ and $G_{h}$ are the vertical and horizontal gradient operators, respectively. Such a loss function allows us to preserve fine details and to remove artefacts.

The feature edge loss $L_{F}$ is defined based on the edge information extracted from a pre-trained VGG-16 network. This design aimed to make the reconstructed image approximate the ground truth from the perspective of the feature edge. It is defined as:

(13) $$L_{F}=\frac{1}{N}\sum_{i=1}^{N}{∥f_{v_{1}}\left(f\left(I_{i}\right),\Theta \right))-f_{v_{1}}\left(J_{i}\right)∥}_{2}+\frac{1}{N}\sum_{i=1}^{N}{∥f_{v_{2}}\left(f\left(I_{i}\right),\Theta \right))-f_{v_{2}}\left(J_{i}\right)∥}_{2},$$

where $f_{v_{1}}$ and $f_{v_{2}}$ are extracted edge features from the first and second layers of the pre-trained VGG-16 network [31]. To show the effectiveness of this loss function, the experiments in super-resolution, image dehazing and other relative fields [24,25] provided sufficient evidence.

## 3. Discussions

Difference from DCPDN: Inspired by the densely connected pyramid dehazing network (DCPDN), we also propose a novel end-to-end RDPN for image dehazing. However, it is worth noting that there are two obvious differences between DCPDN and our model. In general, the sizes of the network models are different. DCPDN uses a U network, a pyramid densely connected network (PDCN) and a GAN to jointly estimate the atmospheric light, the transmission map and the dehazing result simultaneously [24]. Hence, the size of the network model was 268 MB, and the number of network parameters was 12,446,386; while our proposed network used one RDP in the encoder and two RDPs in the decoder, which not only saved GPU memory, but also improved computational efficiency. Using the same platform of PyTorch as that used in DCPCN, the size of the proposed model was 4.3 MB and the number of network parameters was only 787,707, which was only 6% of that used in DCPCN.

Difference from PDCN: The PDCN in DCPDN was designed based on the encoder-decoder structure for estimating the transmission map [24]. Comparing with our proposed RDPN, there are two main differences: First, PDCN uses dense blocks as the basic building blocks, which cannot capture informative texture and structural information for dehazing by stacking dense blocks. In contrast, we used RDP to construct our RDPN, which not only combined the advantages of dense blocks and residual blocks, but also added the multiscale pyramid fusion mechanism in the RDP for learning structural information at different resolutions. Second, PDCN explores the structural information by applying the multilevel pyramid pooling block (MPPB) at the end of the decoder, but ignoring the structural information from intermediate layers. Our RDPN not only learns structural information from each layer of the network by using RDP, but also learns global context information by placing a multilevel pyramid pooling layer (MPPL) at the end of the network.

Difference from RDB: Due to the convincing advantages of the residual dense block (RDB) [29], we propose RDP based on RDB. However, there are three main differences between them. First, RDB was designed for image super-resolution, while our proposed RDP was designed to realize image dehazing. Second, RDB was proposed by combining the advantages of dense blocks and residual blocks, while our proposed RDP added the multiscale pyramid fusion between dense information fusion and residual learning for fully learning the spatial information at different resolutions. Third, existing methods stacked RDBs for extracting features with a fixed scale [29], while we embedded RDP into the encoder-decoder architecture and added a convolution or deconvolution operation following each RDP to realize the down-sampling or the up-sampling operation.

## 4. Implementation Details

The detailed architecture and parameter settings of the RDPN are provided in Table 1, where each RDP in the encoder and decoder has the same setting except the filter number, which depends on the number of output channels from the preceding layer, and these are shown in Table 2. Each convolutional layer in the DIF was followed by a rectified linear unit (ReLU) for improving training efficiency and for adding non-linearity. The growth rate *G* was 32. Adam was selected as the optimization algorithm with a learning rate of $2\times 10^{-3}$ for training the model. The batch size was set as two. Empirical values of λ and β were used, which were 2 and 0.8, respectively. All the training images were resized to 512×512, and the output of the corresponding clear image had three channels (red, green and blue). The RDPN was trained for 3,200,000 iterations.

## 5. Experimental Results

In this section, we further investigate the effectiveness of RDPN. We first introduce our large dataset, which contained both a synthetic dataset and real hazy images for training and testing. Then, we compare our method with several state-of-the-art methods in terms of visual results and accuracy. Finally, a series of analyses and discussion related to the performance, run time and limitations of the RDPN are given.

### 5.1. Datasets

Although there are some existing training datasets, the amount of synthetic hazy images contained in them is enormous. For example, the RESIDE dataset [32] contains 313,950 synthetic outdoor images. Directly using existing datasets for training our model would cost too much training time. Besides, it is also not fair to compare our trained model with other dehazing models that were trained on 4000∼10,000 synthetic images [24]. Therefore, we created our dataset including both indoor and outdoor images. Similar to [24], 1000 depth images from the NYU depth dataset [33] were selected randomly for generating 4000 indoor training images and 400 testing images via Equation (1), with random atmospheric light *A* ∈ {0.5, 1} and scattering coefficient *β* ∈ {0.4, 0.6}. In addition, from the RESIDE dataset [32], another 4000 synthetic training images and an extra 400 test images with β in {0.04, 0.06, 0.08, 0.1, 0.12, 0.16, 0.2} and *A* in {0.8, 0.85, 0.9, 1} were chosen randomly as the outdoor images. Hence, we had 8000 training images and 800 testing images in total, including 400 indoor images denoted as the indoor testing dataset and 400 outdoor images denoted as the outdoor testing dataset.

### 5.2. Testing on the Synthetic Dataset

#### Comparison with Existing Dehazing Methods

In this section, we first compare our model on synthetic datasets (indoor testing dataset and outdoor testing dataset) with six state-of-the-art dehazing methods, including DCP [6], NLP [7], CAP [11], AODN [19], Dehazenet [17] and DCPDN [24]. Two commonly used quality metrics: PSNR and SSIM are used to evaluate the dehazing results. All the PSNR/SSIM measures are reported in Table 3. Compared with other dehazing methods, we see that our proposed RDPN had a higher PSNR and SSIM.

**Figure 4:**
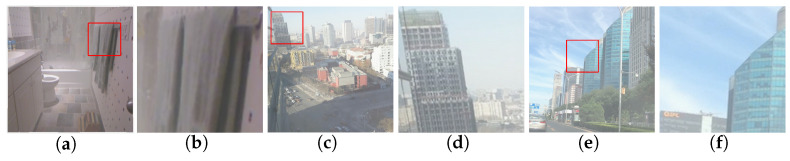
Hazy samples from the synthetic dataset and the corresponding magnified details. (**a**) Hazy Sample 1; (**b**) Magnified detail of hazy Sample 1; (**c**) Hazy Sample 2; (**d**) Magnified detail of hazy Sample 2; (**e**) Hazy Sample 3; (**f**) Magnified detail of hazy Sample 3.

**Figure 5:**
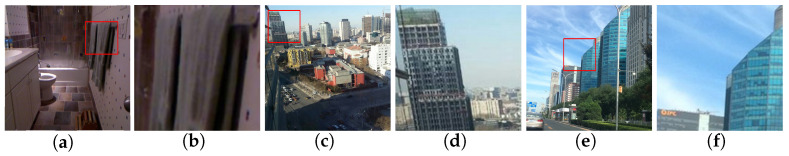
Ground truths of hazy samples and the corresponding magnified details. (**a**) Ground truth of hazy Sample 1; (**b**) Ground truth of the magnified detail of hazy sample 1; (**c**) Ground truth of hazy sample 2; (**d**) Ground truth of the magnified detail of hazy sample 2; (**e**) Ground truth of hazy sample 3; (**f**) Ground truth of the magnified detail of hazy sample 3.

**Figure 6:**
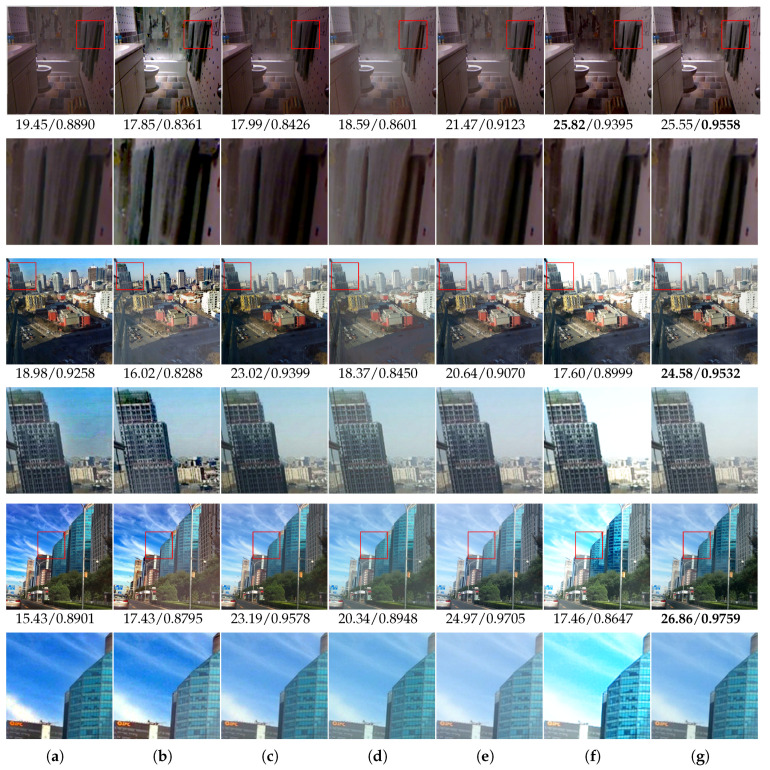
Dehazing results of three samples in the synthetic dataset. (**a**) DCP [6]; (**b**) NLP [7]; (**c**) CAP [11]; (**d**) AODN [19]; (**e**) Dehazenet [17]; (**f**) DCPDN [24]; (**g**) ours.

In Figure 4, three samples with the magnified details from the synthetic dataset were selected for visual comparison. Among them, Figure 4a,c,e are the original hazy images. Figure 4b,d,f are the magnified details of regions enclosed in red rectangles in corresponding hazy samples. The corresponding ground truths of Figure 4 are shown in Figure 5. Meanwhile, Figure 6a–f display the dehazing results of DCP [6], NLP [7], Dehazenet [17], DCPCN [24], CAP [11] and AODN [19], respectively, and the corresponding magnified details are shown in the second, fourth and sixth rows. It can be seen that even though existing methods could remove haze from the original images to some extent, their results tended to be either over dehazed or under dehazed. For example, the results of DCP [6] (Figure 6a) and CAP [11] (Figure 6c) were over dehazed and had some colour distortions, compared with the ground truths (Figure 5), e.g., the towel, building and sky region in magnified images from the second, fourth and sixth rows in Figure 6a,c. In the dehazed results of NLP [7], there were haze residuals and artefacts, which could be observed in the road and tree in the third and fifth rows of Figure 6b. These improperly dehazed results were probably due to the invalid assumption of priors used in the above methods. The AODN and Dehazenet estimates of the dehazing result and the transmission map by neural networks, respectively, could overcome the limitations of the hand-crafted prior based methods, e.g., DCP, NLP and CAP. However, the results shown in Figure 6d,e still contained some hazy residuals. The DCPDN using GAN to optimize the dehazing result estimated by neural networks could obtain clearer results shown in Figure 6f than those of other methods. Unfortunately, upon detailed inspection, this method produced noticeable colour shifts, e.g., the towel in the second row and buildings in the sixth rows. In contrast, our method worked better than others and generated clearer images with less colour distortion. Actually, the results displayed in Figure 6g are visually closest to the ground truth shown in Figure 5. The PSNR/SSIM measures shown under each image also demonstrated the favourable performance of the proposed method.

**Figure 7:**
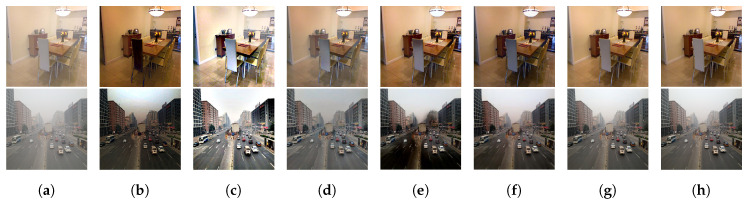
Dehazing results of two samples from the SOTS dataset. (**a**) Input; (**b**) GPE [15]; (**c**) PWAB [13]; (**d**) AMEF [14]; (**e**) GFN [22]; (**f**) EPDN [20]; (**g**) ours; (**h**) ground truths.

RESIDE [32], a recently released dehazing benchmark, was also adopted for further evaluating the performance of RDPN. As a public benchmark for image dehazing and beyond, the sub-dataset SOTS [32] in RESIDE containing 500 indoor images and 500 outdoor images with different haze concentration was used for testing the performance of different dehazing algorithms. The quantitative results of our model and the extra seven state-of-the-art methods tested on SOTS are displayed in Table 4 and Table 5, where the quantitative values of some methods were collected from [20,21,23]. From Table 4, we can see that our model ranked the third among popular dehazing methods on the indoor images of SOTS, only second to the results by GridDN [23] and EPDN [20]. Meanwhile, our model ranked the second on outdoor images of SOTS as shown in Table 5. It is noteworthy that GridDN consisted of a pre-processing module, a dehazing module and a post-processing module for image dehazing. Hence, GridDN had the most competitive performance. In contrast, our model removed haze with one RDP in the encoder and two RDPs in the decoder, with a much simpler architecture and much fewer network parameters. Further, our method even outperformed some recent methods, e.g., RIGAN, GPE, AMEF and GFN. The corresponding dehazing results of two samples from the SOTS dataset are displayed in Figure 7. As can be seen, our results (see Figure 7g) were closest to the ground truth (see Figure 7h), while the results by other methods were either over dehazed or under dehazed (see Figure 7b–f).

### 5.3. Testing on Real Images

**Figure 8:**
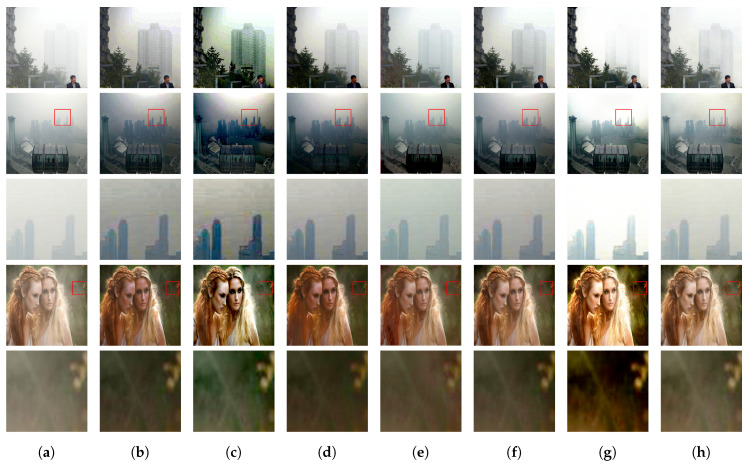
Dehazing results on real-world images downloaded from the Internet. (**a**) Input; (**b**) DCP [6]; (**c**) NLP [7]; (**d**) CAP [11]; (**e**) AODN [19]; (**f**) Dehazenet [17]; (**g**) DCPDN [24]; (**h**) ours. The third row shows the magnified view of the highlighted windows in the second row. The fifth row shows the magnified view of the highlighted windows in the fourth row.

To verify the generalization ability of our model, we further tested RDPN on challenging images provided by previous methods [22,24]. Visual dehazing results produced by RDPN and six state-of-the-art methods are displayed in Figure 8.

The first, second and fourth rows of Figure 8a show three original real-world images. Figure 8b–g display the corresponding results of DCP [6], NLP [7], DCPDN [17], CAP [11] and AODN [19], respectively. Our results are given in Figure 8h. The magnified details of two images using different methods are shown in the third and fifth rows. In Figure 8b–d, the results of DCP [6], NLP [7] and CAP [11] suffered from over dehazed, due to the colour distortion and blocking artefacts shown in Figure 8b–d. The results of AODN [19] and Dehazenet [17] displayed in Figure 8e,f still had some remaining haze in them. Some details shown in the magnified regions were missing, as shown in the third and fifth rows. DCPCN [24] could produce clearer images with strong contrast (see Figure 8g), but part of the buildings in the first, second and third rows was not recovered. In particular, the tops of buildings in the magnified inset shown in the third row of Figure 8g were missing. Furthermore, the magnified region in the fifth row had an over dehazed effect. In contrast, our method could remove haze with visually appealing results in all cases.

### 5.4. Analysis and Discussion

We further analyse and discuss the validity of our RDPN with different network architectures and parameters. Besides, we also discuss the runtime performance and limitations of RDPN.

#### 5.4.1. Different RDP Number

Since the proposed neural network was constructed based on RDP, we first investigated the effect of the number of RDP in the encode and decoder network. To determine the effect of the RDPN’s depth, we trained the network with three different settings: one RDP in the encoder and two RDPs in the decoder (denoted as D=1), two RDPs in the encoder and three RDPs in the decoder (denoted as D=2) and three RDPs in the encoder and four RDPs in the decoder (denoted as D=3). The quantitative comparisons of these three settings are shown in Table 6. We can see that the PSNR and SSIM values of D=1 were higher than those of D=2 and D=3, which demonstrated that stacking more RDPs in the encoder and the decoder would not lead to better performance, as commonly believed. Therefore, we used D=1 as our basic network parameters. Based on the RDPN with D=1, we also investigated the effect of the number of dense convolution layers *C* and the growth rate *G* in DIF. Because the default setting of *C* and *G* in the DIF of RDPN was six and 32, respectively, the settings of C=5, C=7 and G=16, G=64 were adopted for testing the effect of RDPN further. From Table 6, we can see that they produced suboptimal results compared to those of C=6 and G=32.

Generally, it can be seen that RDPN was quite robust based on different configurations and parameter settings, as the results of SSIM in Table 6 ranged between 0.9708 and 0.9752 for both the indoor testing dataset and the outdoor testing dataset. In particular, RDPN with D=1, C=6 and G=32 attained the best performance among the evaluated configurations.

#### 5.4.2. Analysis of the RDP Structure

The proposed RDP for image dehazing is a significant contribution of this paper. To verify its effectiveness, we compared the RDPN with several variants of RDPs. RDP w/o R indicates that the RDP module does not contain the residual learning in the MPF. DRP represents that the residual learning in the original RDP is moved from the end of MPF to the end of DIF. For fair comparison, these network structures (RDPN using RDP w/o R and RDPN using DRP) were the same as the proposed RDPN, except for using different building modules, e.g., RDP w/o R and DRP. As reported in Table 7, the RDPN with the proposed RDP outperformed other models on all datasets, which demonstrated that the design of RDP could take advantage of the dense connection, multi-scale pyramid fusion and residual learning in the best combination.

#### 5.4.3. Different RDP Placement

In our work, we built RDPN with the proposed RDP so that the contextual and structural information from all the layers could be used to obtain more robust features. A question that deserves asking is how the RDP improved the performance of the model? To investigate the effects of using RDP in different layers of the RDPN, we further compared three variant models, namely, RN, RN w/o MPPL and RDPN-decoder, where RN denotes the model with all RDPs in RDPN replaced with dense block (the DIF in the RDP), and RN w/o MPPL means the RN model without MPPL, just as the schematic figures displayed in Figure 3a,b, respectively. RDPN-decoder indicated that in the RDPN model, the RDP in the encoder was replaced with the dense block (the DIF in the RDP), just as the schematic figure shown in Figure 3c. The results reported in Table 7 demonstrate that the performances of these models were inferior to that of the proposed model RDPN. That means the contextual and structural information collected from RDPs in all the layers made contributions to image dehazing. Besides, the performance differences between RDPN-decoder and RDPN were obvious. With the help of RDP inserted in the encoder of RDPN, the output of the encoder could capture more global structural information in the encoding stage and could generate better results with higher PSNR and SSIM values.

#### 5.4.4. Effectiveness of SFEL

To demonstrate the effectiveness of SFEL in the proposed RDPN, we removed the SFEL from RDPN and also set the stride of convolution behind RDP at four to keep a symmetrical feature size in the encoder and decoder. The result of the corresponding model named RDPN w/o SFEL is shown in Table 7. As can be seen, without SFEL degraded the performance of the original model RDPN, as shown in the second row of Table 7. This indicates that SFEL was an important transition layer, which provided rich global information with half of the input image size for deeper stages collecting high level information. Removing SFEL straightforwardly led to missing significant useful global information by shrinking the input feature by a quarter.

#### 5.4.5. The Impact of Regulation Coefficients in the Loss Function

In this work, we adopted the combined loss function of [24] to learn the parameters of the proposed RDPN. That means the feature edge loss $L_{F}$ and gradient loss $L_{G}$ were combined with the common standard $L_{2}$ function in the loss. In Equation (10), $L_{F}$ and $L_{G}$ are multiplied by corresponding weighting coefficients λ and β. To verify the robustness of this loss function, in Table 8, we list results when different settings of weighting coefficients λ and β are considered. As can been seen, using λ=2 and β=0.8, the network obtained the highest PSNR and SSIM values. The other settings lowered the performance in a small range. Hence, the combined loss function had good robustness.

#### 5.4.6. Run Time and Number of Network Parameters

As our network contained three RDPs with significantly fewer parameters than those of other heavy-weight dehazing models, how fast can the proposed method dehaze an image? How many fewer parameters are contained in our dehazing model compared with other methods? In this section, we mainly compare the average run time and number of parameters of the RDPN with the counterparts of several state-of-the-art methods on a computer (Intel Xeon(R) CPU E5-2637 3.5 GHz). Related results are provided in Table 9. Besides, the accuracy of different methods, e.g., average PSNR, obtained by testing them on 500 outdoor images of the public SOTS dataset are also given in Table 9 for comprehensive comparison. From that, we observed that our method ranked second in run time and ranked third in the number of parameters, only second to PIDN and AODN. However, PIDN had fewer parameters, which could be attributed to its use of a recurrent structure to share the parameters in the network. AODN had much fewer parameters and a much shorter run time, because AODN only used five convolutional layers to build the network. However, the design of AODN also led to poor accuracy. From the last row of Table 9, we can see that our method outperformed AODN and most dehazing methods, e.g., DCPDN, PWAB, AMEF and GPE, on outdoor images of the SOST dataset in terms of PSNR by up to 4.25 dB at least, only second to PIDN [20]. Hence, our method was much more efficient in comparison (the run time was reduced to 0.021 s) and could produce better results with fewer parameters (the number of parameters was 1,534,799, only 6% of that used by DCPDN) than these state-of-the-art methods.

#### 5.4.7. Limitations

**Figure 9:**
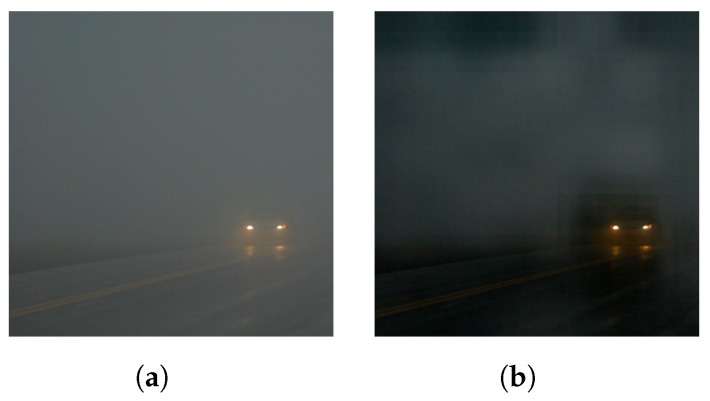
The failed case for RDPN; (**a**) Night-time image; (**b**) Dehazing image.

The training outdoor images were taken during the daytime and synthesized with white fog. Therefore, the model did not hold for images taken in the evening or at night with strong grey smog. Figure 9 shows that the RDPN was not able to produce a clear image (see Figure 9a) for a night-time image (see Figure 9b). This was probably because the training dataset did not contain similar images, resulting in the RDPN model failing to learn the corresponding mapping function. We plan to address this problem by adding more comprehensive outdoor hazy images taken at different times into the training dataset.

## 6. Conclusions

In this paper, we presented a novel end-to-end residual dense pyramid network (RDPN) based on the encoder and decoder architecture for image dehazing, where the proposed residual dense pyramid (RDP) served as the basic building module. RDP used multiscale pyramid fusion (MPF) to learn spatial information, leading to effective information fusion. After using one RDP in the encoder and two RDPs in the decoder in RDPN, the proposed framework also adopted a pyramid pooling module to capture the global content information from different scales before the final mapping. Extensive experiments showed that the average PSNR of the proposed RDPN was 26.82 dB, which outperformed most art-of-the-state methods, e.g., the recent densely connected pyramid dehazing network, all-in-one dehazing network, enhanced pix2pix dehazing network, pixel-based alpha blending, artificial multi-exposure image fusions and genetic programming estimator, by up to 4.25 dB. Besides, the run time of the RDPN was reduced to 0.021 s, and the number of parameters in the network was 1,534,799, which was only 6% of that used by the densely connected pyramid dehazing network. Hence, RDPN achieved superior performance over state-of-the-art methods with a significantly smaller model size and much fewer network parameters.

## Figures and Tables

**Table 1:** Detailed configuration of the proposed RDPN. SFEL, shallow feature extraction layer; MPPL, multilevel pyramid pooling layer; GIFL, global information fusion layer.

Name	Layer Type	Kernel	Filter	Stride	Pad
SFEL	1 convolution	1 × 1	32	2	0
Encoder	RDP 1 convolution	/ 1 × 1	32 64	/ 2	/ 0
Decoder	RDP 1 deconvolution RDP 1 deconvolution	/ 3 × 3 / 3 × 3	64 32 64 16	/ 2 / 2	/ 1 / 1
MPPL	4 poolings 4 up-samplings 4 convolutions	4 × 4, 8 × 8, 16 × 16, 32 × 32 / 1 × 1	16 / 1	4,8,16,32 / 1	0 / 0
GIFL	1 convolution	3 × 3	3	1	1

**Table 2:** Detailed configuration of the proposed RDP. DIF, dense information fusion; MPF, multiscale pyramid fusion; RL, residual learning.

Name	Layer Type	Kernel	Filter	Stride	Pad
DIF	6 convolutions 1 convolution	3 × 3 1 × 1	32 / 64 32 / 64	1 1	1 0
MPF	4 poolings 4 up-samplings 4 convolutions 1 convolution	2 × 2, 4 × 4, 8 × 8, 16 × 16 / 1 × 1 1 × 1	32 / 64 / 1 32 / 64	2,4,8,16 / 1 1	0 / 0 0
RL	summation	/	32 / 64	/	/

**Table 3:** Quantitative comparisons on the synthetic testing dataset in terms of PSNR/SSIM. The best results are in bold. DCP, dark channel prior; NLP, non-local prior; CAP, colour attenuation prior; AODN, all-in-one dehazing network; DCPDN, densely connected pyramid dehazing network.

	DCP [6]	NLP [7]	CAP [11]	AODN [19]	Dehazenet [17]	DCPDN [24]	Ours
Indoor testing dataset	13.97/0.8842	17.44/0.7959	18.04/0.8567	17.83/0.8842	20.19/0.8773	29.22/0.9560	**29.29/0.9747**
Outdoor testing dataset	13.59/0.8664	16.59/0.7736	16.01/0.7696	18.54/0.852	22.30/0.9159	28.12/0.9416	**28.59/0.9752**

**Table 4:** Quantitative comparisons on the indoor images of the SOTS in terms of PSNR/SSIM. Red, green and blue indicate the best, second best and third best performance, respectively. GPE, genetic programming estimator; PWAB, pixel-wise alpha blending method; AMEF, artificial multi-exposure image fusion; GFN, gated fusion network; GridDN, GridDehazeNet; EPDN, enhanced pix2pix dehazing network; RIGAN, generative adversarial networks with residual inception module.

GPE [15]	PWAB [13]	AMEF	GFN [22]	GridDN [23]	EPDN [20]	RIGAN [21]	Ours
11.97/0.6301	15.96/0.7415	16.01/0.7573	22.30/0.8800	32.16/0.9836	25.06/0.9232	18.61/0.8179	19.66/0.8972

**Table 5:** Quantitative comparisons of the outdoor images of the SOTS in terms of PSNR/SSIM. Red, green and blue indicate the best, second best and third best performance, respectively.

GPE [15]	PWAB [13]	AMEF [14]	GFN [22]	GridDN [23]	EPDN [20]	Ours
15.91/0.7297	12.33/0.6759	17.62/0.8201	21.55/0.8444	30.86/0.9819	22.57/0.8630	26.82/0.9598

**Table 6:** Ablation study: quantitative PSNR/SSIM for different configurations of the proposed networks.

	RDPN, (*C* = 6, *G* = 32)			RDPN (*D* = 1)		RDPN (*D* = 1)	
	*D* = 1	*D* = 2	*D* = 3	*C* = 5	*C* = 7	*G* = 16	*G* = 64
Indoor testing dataset	29.29/0.9747	28.69/0.9675	29.09/0.9713	29.02/0.9710	28.84/0.9708	29.05/0.9721	29.11/0.9729
outdoor testing dataset	28.59/0.9752	28.28/0.9710	28.42/0.9729	28.57/0.9733	28.30/0.9725	28.35/0.9723	28.41/0.9739

**Table 7:** Performance of RDPN and its variant models in terms of PSNR/SSIM. Best results in bold.

Model	Indoor Testing Dataset	Outdoor Testing Dataset
RDPN	**29.29/0.9747**	**28.59/0.9752**
RDPN using RDP w/o R	28.78/0.9642	27.89/0.9701
RDPN using DRP	29.10/0.9732	28.32/0.9720
RN	28.84/0.9251	27.15/0.9111
RN w/o MPPL	27.96/0.9133	27.01/0.9087
RDPN-Decoder	28.97/0.9697	27.77/0.9712
RDPN w/o SFEL	23.04/0.9274	25.50/0.9568

**Table 8:** Analysis of the loss function in terms of PSNR/SSIM when different weighting coefficients are used. Best results in bold.

Setting	Indoor Testing Dataset	Outdoor Testing Dataset
*λ* = 2, *β* = 0.8	**29.29/0.9747**	**28.59/0.9752**
*λ* = 0.8, *β* = 2	29.22/0.9745	28.54/0.9749
*λ* = 1, *β* = 1	29.26/0.9746	28.55/0.9750

**Table 9:** Comparison of the proposed RDPN with other state-of-the-art methods in terms of run time, number of parameters and accuracy. PIDN was re-trained with our synthetic hazy images. Average PSNR values and run time are reported on the outdoor images of the public SOTS dataset. Red, green and blue indicate the best, second best and third best results, respectively.

	PIDN [20]	PWAB [13]	AMEF [14]	GPE [15]	AODN [19]	DCPDN [24]	Ours
Platform	PyTorch	MATLAB	MATLAB	Python	PyTorch	Python	PyTorch
Run time	0.115	0.46	1.67	2.46	0.002	0.056	0.021
Parameters	172,044	/	/	/	1761	12,446,386	1,534,799
accuracy	26.93	12.33	17.62	15.91	20.29	19.93	26.82
